# Cardiomyocyte-fibroblast crosstalk in the postnatal heart

**DOI:** 10.3389/fcell.2023.1163331

**Published:** 2023-04-03

**Authors:** Maria Uscategui Calderon, Brittany A. Gonzalez, Katherine E. Yutzey

**Affiliations:** ^1^ Division of Molecular Cardiovascular Biology, The Heart Institute, Cincinnati Children’s Medical Center, Cincinnati, OH, United States; ^2^ Department of Pediatrics, University of Cincinnati College of Medicine, Cincinnati, OH, United States

**Keywords:** cardiomyocyte, cardiac fibroblast, postnatal heart, extracellular matrix, cell signaling, heart regeneration

## Abstract

During the postnatal period in mammals, the heart undergoes significant remodeling in response to increased circulatory demands. In the days after birth, cardiac cells, including cardiomyocytes and fibroblasts, progressively lose embryonic characteristics concomitant with the loss of the heart’s ability to regenerate. Moreover, postnatal cardiomyocytes undergo binucleation and cell cycle arrest with induction of hypertrophic growth, while cardiac fibroblasts proliferate and produce extracellular matrix (ECM) that transitions from components that support cellular maturation to production of the mature fibrous skeleton of the heart. Recent studies have implicated interactions of cardiac fibroblasts and cardiomyocytes within the maturing ECM environment to promote heart maturation in the postnatal period. Here, we review the relationships of different cardiac cell types and the ECM as the heart undergoes both structural and functional changes during development. Recent advances in the field, particularly in several recently published transcriptomic datasets, have highlighted specific signaling mechanisms that underlie cellular maturation and demonstrated the biomechanical interdependence of cardiac fibroblast and cardiomyocyte maturation. There is increasing evidence that postnatal heart development in mammals is dependent on particular ECM components and that resulting changes in biomechanics influence cell maturation. These advances, in definition of cardiac fibroblast heterogeneity and function in relation to cardiomyocyte maturation and the extracellular environment provide, support for complex cell crosstalk in the postnatal heart with implications for heart regeneration and disease mechanisms.

## 1 Introduction

The mammalian heart is composed of multiple cell types including cardiomyocytes, fibroblasts, endothelial cells, macrophages, and smooth muscle cells (Pinto et al., 2016). Over the past few years, the development of new sequencing technologies has facilitated more accurate identification of cardiac cell populations and their specific roles during development, disease, and homeostasis (Borg et al., 1981; Hortells et al., 2020; Wang et al., 2020). Importantly, after birth, the heart adapts to the substantial increase in its physiological demands by increasing its size and volume with thickening of the ventricular wall and increased muscular tensile strength (Günthel et al., 2018). Notably, the organized function of these different cell populations, in combination with the complex network of intercellular circuits of communication, is essential to maintain a healthy heart and its disruption leads to cardiovascular disease conditions.

Cardiomyocytes are the muscle cells of the heart, and their synchronous contraction is required to pump blood through the body. They are among the most energetically demanding cells as they continuously contract throughout an individual’s lifespan. Thus, their cytoplasm is packed with sarcomeres, the contractile units of muscle cells, and mitochondria, which are needed to satisfy their high energy requirements. During the postnatal period (P0–P30), cardiomyocytes undergo changes in size, oxidative capacity, and energy production to support the changing physiological needs of the growing heart and body (Borg et al., 1981). At the same time, cardiomyocytes undergo increased ploidy (binucleation), cell cycle arrest, and transition to hypertrophic growth (Li et al., 1996; Soonpaa et al., 1996; Hirai et al., 2016). These maturation events lead to specialization of contractile properties, as well as determine the differential numbers of cardiomyocytes in the atria and ventricles linked to physiological needs of each chamber (Bergmann et al., 2015; Tucker et al., 2020).

Cardiac fibroblasts are considered the most abundant non-cardiomyocyte cell type of the heart (Tallquist and Molkentin, 2017). Located within the myocardial interstitium and in proximity to capillaries and blood vessels, they have critical functions in extracellular matrix (ECM) deposition, maintenance, and remodeling. Mature cardiac fibroblasts are characterized by an elongated, spindle-like morphology with a granular cytoplasm and an extensive rough endoplasmic reticulum (Ivey and Tallquist, 2016). Cardiac fibroblast numbers change with development, aging, and disease. In rats and mice, the number of fibroblasts doubles postnatally from 30% to 64% in rats and from 10% to 25% in mice until full maturity is reached in adulthood (Banerjee et al., 2007). Moreover, single cell RNA sequencing (scRNAseq) has led to the identification of several subpopulations of cardiac fibroblasts within the heart, which each display their own molecular and structural characteristics (Tucker et al., 2020). Therefore, it is not surprising that cardiac cell populations go through defined transitions and specialization after birth. Importantly, the increase in cardiac fibroblasts during the period when cardiomyocytes cease to proliferate and transition to hypertrophic growth highlights the important relationship between these cells during the postnatal period.

Cellular crosstalk is active in the postnatal period, when cardiomyocytes undergo cell cycle arrest simultaneously with a two-fold increase in the number of cardiac fibroblasts (Anversa et al., 1980; Pinto et al., 2016). Along with the increase in number of cardiac fibroblasts, the ECM undergoes remodeling with synthesis and reorganization of the matrix to be able to withstand the forces that are generated postnatally in the ventricles (Bildyug, 2021). Neonatal cardiomyocytes can adhere to ECM components of either the basement membrane or the interstitial matrix and these connections influence their development (Bowers et al., 2010). Importantly, the microenvironment of the postnatal heart offers clues to understanding the importance of specific crosstalk mechanisms that underlie fibroblast and cardiomyocyte maturation, which to date remain relatively unknown in comparison with extensive information available on these cells in response to adult cardiac injury.

## 2 Cardiomyocyte and fibroblast development

### 2.1 Embryonic origins of cardiomyocytes and fibroblasts

During prenatal heart development, cardiac fibroblasts and cardiomyocytes originate from distinct populations of mesodermal progenitors of the vertebrate embryo. Cardiomyocytes and endocardial endothelial cells are among the first differentiated cell types arising from the anterior lateral plate mesoderm early in embryogenesis (Evans et al., 2010). The heart forms initially as a tube consisting of an endothelial cell layer surrounded by differentiated beating cardiomyocytes. At the venous pole of the looped heart, the proepicardium is induced and migrates over the surface of the heart to form the epicardium. Epicardial-derived cells (EPDCs) are generated through an epithelial-to-mesenchymal transition and include progenitors of coronary vascular smooth muscle and cardiac fibroblasts (Acharya et al., 2012; Braitsch et al., 2012; Braitsch and Yutzey, 2013; Moore-Morris et al., 2014a). In addition, secreted signals from the epicardial cell layer, including Insulin-like growth factor (IGF)2, promote fetal cardiomyocyte proliferation and maturation in the developing ventricles of mice (Li et al., 2011). Differentiation of EPDCs into cardiac fibroblasts is dependent on retinoic acid signaling and the basic helix-loop-helix (bHLH) transcription factor Tcf21, which is a critical transcriptional regulator of the fibroblast lineage in the developing, mature, and diseased heart (Acharya et al., 2012; Braitsch et al., 2012). Longitudinal cell fate analysis using lineage-restricted Cre drivers in mice demonstrates that the majority of mature cardiac fibroblasts arise from epicardial progenitors (Tbx18Cre+), with additional contributions from endocardial endothelial cells (Tie2Cre+) predominantly in the interventricular septum (Ali et al., 2014; Moore-Morris et al., 2014b; Sereti et al., 2018). Differentiated cardiac fibroblasts are present prenatally as indicated by Collagen1a1 mRNA expression (Braitsch et al., 2012) and transgenic reporters (Moore-Morris et al., 2014b), but this population undergoes extensive expansion, activation and maturation during the postnatal period.

### 2.2 Cardiomyocyte maturation (postnatal days P0-P30)

*In vivo*, cardiomyocytes undergo various adaptive functional, structural, and metabolic changes during the postnatal period (P0-P30), which has been extensively reviewed elsewhere (Padula et al., 2021). The intense study of cardiomyocyte maturation is of interest for cardiac regenerative medicine since murine cardiomyocytes have the capacity to regenerate through proliferation of existing cardiomyocytes prior to and soon after birth (Senyo et al., 2013). However, approximately a week after birth, cardiomyocyte maturation factors, including exposure to atmospheric oxygen (Puente et al., 2014) and thyroid hormone (Chattergoon et al., 2012; Hirose et al., 2019), induce cardiomyocyte cell cycle arrest and, thus, are antagonistic to cardiac regeneration. During the perinatal period (embryonic day E) 18.5 - postnatal day (P) 7, cardiomyocytes undergo structural changes as they shift from round, small cells with disorganized contractile structures, to large, rod-shaped cells with highly organized myofibrils (Yang et al., 2006). Between P3 and P7 in mice, cardiomyocytes undergo karyokinesis in the absence of cytokinesis, which leads to multinucleation and increased nuclear ploidy, followed by cell cycle arrest and loss of regenerative capacity by P10, although strain-dependent differences in timing have been observed (Leu et al., 2001; Porrello et al., 2011; Patterson et al., 2017; Hesse et al., 2018). In zebrafish, the lifelong capacity for cardiac regeneration has been linked to the prevalence of mononucleated diploid cardiomyocytes since perturbations in cytokinesis and increased ploidy leads to loss of their regenerative potential (Patterson et al., 2017; González-Rosa et al., 2018). Thus, cardiomyocyte proliferation and ploidy status have important implications for heart regeneration and growth capacity.

Also, during the postnatal period, sarcomeric proteins including myosin heavy chains and cardiac troponins, switch from embryonic to adult isoforms to enable a more efficient contraction necessary to sustain the growing physiological needs of the heart (Siedner et al., 2003; Yin et al., 2015; Padula et al., 2021). Further, cardiomyocyte maturation and specialization results in increased contractility subject to multiple physiologic and hormonal cues in the postnatal period. Communication of cardiomyocytes amongst themselves and with other cell types in the heart are affected by electrophysiological coupling and maturation of ion channels. Moreover, maturation of cell-cell and ECM-cell interactions also are dynamically regulated during the first week after birth (Wu et al., 2002). In cardiomyocytes, adaptive metabolic changes depend on mitochondrial remodeling that begins embryonically at around E13.5 (Drenckhahn, 2011), with significant increases in mitochondrial numbers, size and distribution in the perinatal period. Importantly, this internal organization is required for proper function to sustain the growing cardiac demand (Dorn et al., 2015). Additionally, from P0 to P7, cardiomyocytes undergo metabolic substrate transition from being primarily glycolytic to being fully dependent on oxidative phosphorylation and utilizing fatty acids as their main energy source (Lopaschuk et al., 2010). Finally, T-tubules, which are invaginations in the plasma membrane that breach transversely into the center of mature cardiomyocytes to facilitate calcium movement through the large cell, form at P6-P15, facilitating efficient excitation-contraction coupling (Tibbits et al., 2002). Together, these features of cardiomyocyte maturation in the postnatal period are critical for adult cardiac structure and function.

### 2.3 Fibroblast heterogeneity and changes in fibroblast activity from P0-P30

**FIGURE 1:**
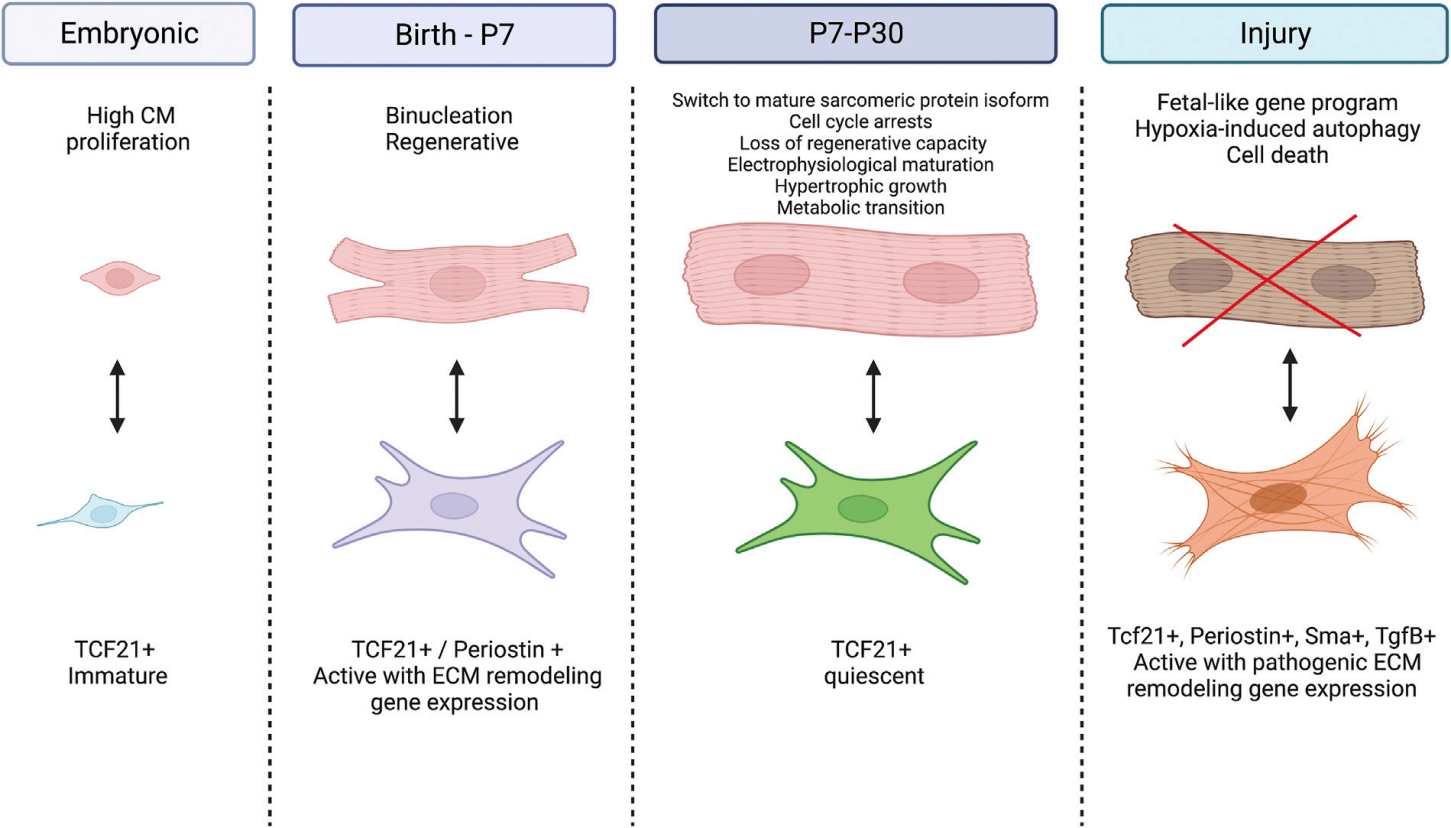
Cardiomyocyte-Fibroblast cellular crosstalk in the developing and diseased heart. During the embryonic period, cardiac fibroblasts remain immature while cardiomyocytes experience high rates of proliferation. At birth, fibroblasts become activated and signal for cardiomyocytes to exit the cell cycle, switch to hypertrophic growth and undergo metabolic transition. Between P7 and P30, fibroblasts return to their quiescent state while cardiomyocytes further their maturity by switching their expression to adult sarcomeric proteins and mature electrophysiology. After injury, fibroblasts become activated and secrete pathogenic ECM proteins while cardiomyocytes experience hypoxia induced autophagy and eventually cell death. Created with BioRender.com.

Cardiac fibroblasts are the primary cell type responsible for deposition of the ECM that occurs in the postnatal period. In the mature heart, cardiac fibroblasts are relatively quiescent but during injury and development they actively contribute to tissue remodeling by synthesizing ECM and secreting factors that interact with neighboring cells including cardiomyocytes. Cardiac fibroblast contributions to the pathologic fibrotic response after cardiac injury and in heart failure have been extensively studied and previously described in other reviews (Baum and Duffy, 2011; Travers et al., 2016). Much less is known of normal cardiac fibroblast function and contributions to postnatal heart development *in vivo*. Here, we focus on the role of these cardiac fibroblasts in generating the normal fibrous matrix of the heart and influencing maturation of cardiomyocytes in the postnatal period (Figure 1).

Cardiac fibroblasts are heterogeneous in development and disease in terms of cell origins and expression of specific ECM components (Kanisicak et al., 2016; Moore-Morris et al., 2018). Recent cell sorting and sequencing studies demonstrated active changes in fibroblast populations and maturation status during postnatal development. Ivey et al. reported non-myocyte proliferation rates peak in the first week of postnatal growth, similar to the timing of cardiomyocyte cell cycle arrest, which is followed by decreases in proliferation to adult quiescent levels by 3 weeks of age (Ivey et al., 2018). Similarly, cardiac fibroblasts marked by Tcf21Cre-driven reporter expression are actively proliferating in the postnatal period (P3-P7), supporting parallel fibroblast and cardiomyocyte maturation dynamics (Hortells et al., 2020). Comparison of fibroblasts at the different stages of development had initially revealed at least two distinct types of neonatal fibroblasts in the heart (Misra et al., 2021). One of these is linked to ECM structure/organization and another with regulation of cell migration (Wang et al., 2019). Additional scRNAseq reported five different fibroblast populations with distinct genetic gene expression states, implicated in ECM remodeling, cell migration and vascular remodeling (Wang et al., 2020). Periostin-expressing (periostin+) fibroblasts are important for scar formation after injury in adults, and during early postnatal development, a transient subpopulation of periostin+ fibroblast is present at P3-P7 (Kanisicak et al., 2016; Hortells et al., 2020). Additional evidence for neonatal cardiac fibroblasts heterogeneity is provided by differential expression of Tcf21+ and periostin+ cell populations as indicated by reporter genes (Hortells et al., 2020). Overall, recent sequencing technologies have revealed unexpected fibroblast diversity in the postnatal heart, however further validation of differential functions in heart maturation and physiology *in vivo* are needed.

In the adult heart, fibroblasts are quiescent and, for the most part, reside in the cardiac interstitium or surround coronary vessels. As a response to cardiac injuries, such as cardiomyopathy or ischemia, resident fibroblasts become activated and further differentiate into additional dynamic states that mediate acute wound healing and long-term tissue remodeling with scarring (Moore-Morris et al., 2014a; Kanisicak et al., 2016; Fu et al., 2018). Importantly, transforming growth factor beta (Tgfβ) signaling and induction of alpha-smooth muscle actin (αSMA) in cardiac myofibroblasts exemplify the pathological fibrotic state. Interestingly, Tgfβ signaling and αSMA activation are not prevalent in neonatal cardiac fibroblast sequencing profiles, supporting different molecular mechanisms of physiological and pathological cardiac remodeling and ECM production (Wang et al., 2019; Hortells et al., 2020; Wang et al., 2020). Further, markers specific to developmental, quiescent, activated, and pathologic states of cardiac fibroblasts may be informative in investigating the contributions of fibroblasts to heart maturation or disease responses.

### 2.4 Molecular and cellular crosstalk in the postnatal heart

The specific crosstalk mechanisms between cardiac fibroblasts and myocytes in the developing heart have recently begun to be studied by selective cell ablation studies in genetic mouse models soon after birth. Ablation of periostin (Postn)-Cre expressing cells at P1-P3 inhibits cardiomyocyte maturation, as indicated by the persistence of small mononucleated myocytes expressing embryonic contractile protein isoforms (Hortells et al., 2020). Similarly, ablation of platelet-derived growth factor receptor alpha (Pdgfrα)Cre expressing fibroblasts also results in decreased tissue stiffness and increased cardiomyocyte mitotic index postnatally (Kuwabara et al., 2022a). In adults, ablation of PdgfrαCre or PostnCre-expressing cardiac fibroblasts prevents severe scarring after myocardial injury (Kanisicak et al., 2016; Kuwabara et al., 2022b). Thus, cardiac fibroblasts, generated during development, differentiate into dynamic states in response to context dependent stimuli with differential responses related to ECM production and crosstalk with cardiomyocytes (Figure 1).

Additional support for the importance of cardiac fibroblasts in inducing cardiomyocyte maturation is provided by coculture experiments of human induced pluripotent stem cell (iPSC) derived cardiomyocytes and fibroblasts. In these studies, the presence of non-cardiomyocyte cells enhances cardiomyocyte maturation through direct physical interactions, as well as through paracrine molecules that are secreted from fibroblasts and act on cardiomyocytes (Kim et al., 2010; Yoshida et al., 2018). Further, scRNAseq of mouse hearts from several developmental stages identified regulatory signaling networks of different cell types and their interactions, providing further evidence that cardiac fibroblasts influence cardiomyocyte maturation through changes in their transcriptional program that includes ECM maturation (Wang et al., 2020; Wang et al., 2022). However, the exact mechanisms by which fibroblasts communicate with cardiomyocytes remain to be determined.

### 2.5 Signaling pathways involved in cardiomyocyte and fibroblast crosstalk

Cardiomyocyte and fibroblast maturation in the postnatal heart are controlled by multiple signaling pathways including Wnt/β-catenin and Hippo/Yap. Further, these major signaling pathways, together with Tgfβ signaling, crosstalk with each other to direct different cellular responses to cardiac injury. However, their specific roles during the postnatal window of heart development are not fully understood.

In mammals, the Wnt/β-catenin signaling pathway is involved in embryonic heart development, including regulation of cardiomyocyte proliferation. In early embryos, inhibition of Wnt/β-catenin signaling is required for vertebrate heart specification, which is revealed by the formation of ectopic hearts after conditional inactivation of *ß*-catenin in the definitive endoderm of the mouse embryo (Lickert et al., 2002). These findings are highly relevant for efforts to produce human cardiomyocytes for therapeutic transplantation from embryonic stem cells (ESCs) or iPSCs in culture. Further, studies in zebrafish, mouse embryos, and in mouse and human embryonic stem cells have identified temporally distinct roles for Wnt/β-catenin signaling during vertebrate heart development (Ueno et al., 2007; Gessert and Kühl, 2010). For example, upregulation of Wnt signaling *in vitro* results in significant increases in proliferation of mature cardiomyocytes in mice and human iPSC-derived cells (Fan et al., 2018). During the postnatal period, *ß*-catenin regulates cardiomyocyte proliferation and metabolic maturation (Quaife-Ryan et al., 2020; Balatskyi et al., 2021; Olcum et al., 2022). Treatment of neonatal or adult rat cardiomyocytes *in vitro* with Wnt inhibitory small molecule BIO induces cell proliferation and mitosis (Tseng et al., 2006). Remarkably, during neonatal heart regeneration, intercellular signaling from cardiomyocytes to cardiac fibroblasts occurs *via* non-canonical Wnt signaling (Misra et al., 2021). However, the source of Wnt ligands during normal postnatal development, intersecting signaling mechanisms, and the specific cells involved are not well-defined.

The Hippo signaling pathway is a critical regulator of cardiac growth during development. Embryonically, the Hippo pathway downstream effector Yap is required for cardiomyocyte proliferation (Ai et al., 2007), and when activated, is sufficient to promote the proliferation of postmitotic cardiomyocytes (von Gise et al., 2012). At birth, Hippo signaling becomes activated to prevent cell cycle re-entry of cardiomyocytes by inhibiting the transcriptional activity of Yap and preventing its translocation into the nucleus. Instead, Hippo pathway activation promotes Yap degradation in the cytoplasm (Xiao et al., 2016) and thus, limits heart size. Interestingly, postnatal cardiomyocyte-specific deletion of Yap resulted in elevated cardiomyocyte apoptosis and dilated cardiomyopathy in mice (Del Re et al., 2013); thus, complete loss of Yap function leads to insufficient cardiac hypertrophy and cardiac dysfunction. This suggests Hippo signaling likely does not completely shut down Yap function after birth, but this has not been directly tested. In adult postmitotic cardiomyocytes, Yap suppresses the Wnt/β-catenin pathway through the direct binding to disheveled or *ß*-catenin (Varelas et al., 2010; Barry et al., 2013) and promotes the expression of genes associated with prenatal heart development (Heallen et al., 2011). However, the cellular origins of Hippo pathway induction and potential crosstalk interactions among multiple cell types in the postnatal heart remains unknown.

Relatively little information is available on paracrine signaling by cardiac fibroblasts in the postnatal heart. In the adult heart after injury, Tgfβ signaling has well-established roles in the cardiac fibrotic response, including induction of pathologic activation of cardiac fibroblasts, and is the driving force for myofibroblast differentiation (Khalil et al., 2017). However, which cells secrete Tgfβ isoforms responsible remains unknown. Importantly, Tgfβ pathway stimulation results in cardiomyocyte hypertrophy and electrophysiological changes (Cartledge et al., 2015; Popek et al., 2022), but the potential role for Tgfβ signaling in activation of fibroblasts and ECM production during the postnatal period has not been defined. Evidence for expression of other secreted ligands in postnatal cardiac fibroblasts is provided by RNAseq datasets. For example, gene expression related to Bone morphogenetic protein (Bmp) and Wnt signaling, are more prevalent during the postnatal period (Hortells et al., 2020; Wang et al., 2020). However, additional studies need to be done to identify specific functions of these signaling pathways and potential paracrine signaling from fibroblasts in the postnatal heart.

## 3 Evolution and conservation of cardiomyocyte-fibroblast crosstalk

### 3.1 Zebrafish

The mature zebrafish heart is notable in its capacity to fully regenerate with generation of new cardiomyocytes after injury without forming a scar (Tzahor and Poss, 2017). This is in contrast to mammalian hearts that lose their ability to regenerate soon after birth and undergo scarring after injury (Porrello et al., 2011). In the developing zebrafish heart, Tcf21+ epicardial-derived cells promote cardiomyocyte proliferation and myocardial growth through fibroblast growth factor (FGF) and vascular endothelial growth factor (VEGF) signaling (Boezio et al., 2023). In adult zebrafish hearts after injury, cardiac fibroblasts are activated, express periostin and are required for cardiomyocyte proliferation and regeneration (Sánchez-Iranzo et al., 2018). More recent, scRNAseq analysis has identified a subpopulation of pro-regenerative cardiac fibroblasts and have implicated Wnt signaling as a regulator of fibroblast activation critical for regeneration in the injured heart (Hu et al., 2022). Interestingly, ablation of Wnt signaling in adult cardiac fibroblasts also prevents hypertrophy and improves cardiac outcomes after pressure overload in mice (Xiang et al., 2017). Together these findings support multiple cardiomyocyte-fibroblast crosstalk mechanisms in zebrafish heart development and injury response. Some of these mechanisms are conserved in mammals and could provide insights into potential therapeutic avenues to promote regenerative repair after cardiac injury.

### 3.2 Pigs

In mammals, cardiomyocytes undergo maturation including bi/multinucleation of cardiomyocytes and transition to mature contractile protein gene expression in parallel with cardiac fibroblast proliferation and generation of the fibrous ECM of the myocardium. While the specific number of nuclei, timing of cell cycle arrest, and transition to hypertrophic growth are not identical among mammalian species, the postnatal period represents a period of dynamic changes and increased cardiac output in multiple species (Velayutham et al., 2019). For example, porcine cardiomyocytes become progressively multinucleated with longitudinal growth 1–2 months after birth, but proliferation of mononuclear diploid cardiomyocytes ceases in the week after birth, along with loss of cardiomyocyte regenerative capacity (Ye et al., 2018; Zhu et al., 2018; Velayutham et al., 2020). At the same time, ECM gene expression, collagen fiber formation, and indicators of activated fibroblasts, such as periostin, are prevalent in the 2 weeks after birth, similar to rodents (Velayutham et al., 2020). However, cardiomyocyte-fibroblast crosstalk mechanisms and potential contributions to cardiac regenerative capacity have not been extensively examined in pigs, and conservation of previously identified crosstalk mechanisms has not yet been determined.

### 3.3 Humans

Much less is known of molecular and cellular mechanisms of cardiomyocyte-cardiac fibroblast crosstalk and heart maturation in human infant hearts. Similar to other mammals, human infant cardiomyocytes undergo multinucleation and polyploidization after birth. Cytokinesis failure related to downregulation of Ect2 has been implicated in bi/multinucleation of postnatal human cardiomyocytes (Liu et al., 2019). Anecdotally, cardiac injury in human infants has been hypothesized to lead to regenerative repair, but conditions for assessment of human cardiac regeneration are rare and there have been no follow-up reports since the original 2016 publication (Haubner et al., 2016). Even less information is available about human infant cardiac fibroblasts or potential crosstalk mechanisms with cardiomyocytes. Single cell/nuclear RNA sequencing analysis of fetal human hearts supports fibroblast diversity and activation in the developing heart (Asp et al., 2019; Mehdiabadi et al., 2022). Less is known of neonatal human heart populations and interactions, but single cell analyses may be informative here.

Additional studies of human iPSC provide support for conserved mechanisms in epicardial origins and fibroblast diversification (Witty et al., 2014). Interestingly human tissue engineering demonstrates that the presence of cardiac fibroblasts supports maturation and increased contractility of cardiomyocytes, further supporting the critical role of cardiac fibroblasts in terminal maturation of cardiomyocytes in different contexts (de Lange et al., 2021). The specific cardiomyocyte-cardiac fibroblast crosstalk mechanisms were not identified in this study, but they could be relevant for understanding of neonatal cardiac maturation events.

## 4 The extracellular matrix of the heart

**FIGURE 2:**
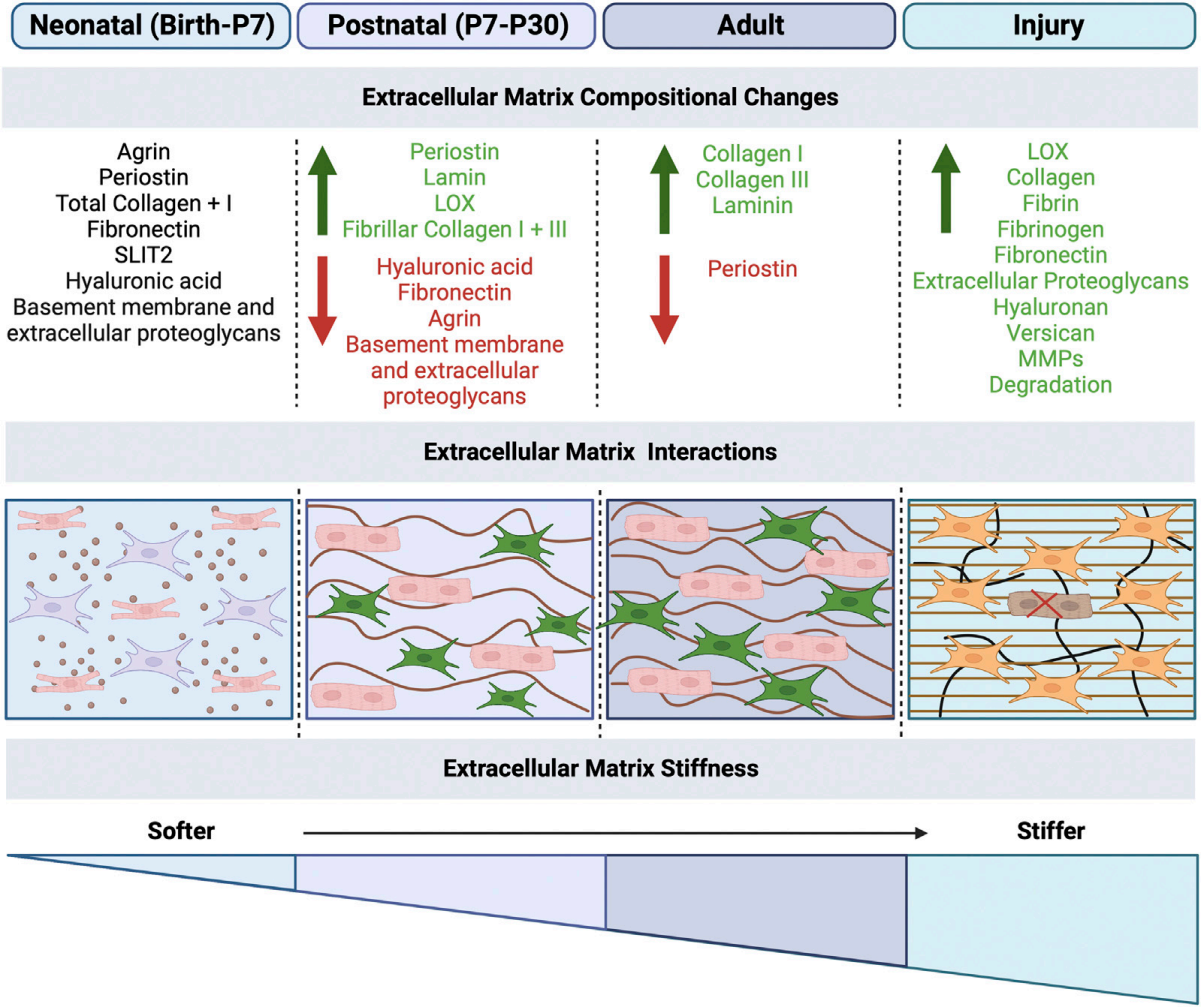
Extracellular matrix mechanical effects on cardiomyocytes and fibroblasts in development and disease. The extracellular matrix (ECM) plays a role in the maturation of cardiomyocytes and function of fibroblasts. After birth, the ECM undergoes mechanical changes, specifically the increased stiffness of the matrix due to changes in the ECM protein composition, secreted by fibroblasts. ECM stiffness increases from neonatal to adulthood, with the highest stiffness occurring during disease, which results in fibrosis and scarring. The compositional changes that occur at each stage are listed, with green as increase and red as decrease in ECM proteins. Created with BioRender.com.

The cardiac ECM is composed of collagen, elastin and proteoglycans, providing structural support for cells as well as a platform for cell-cell interaction, proliferation, and migration (Bowers et al., 2010). The cardiac ECM also is involved in mechanical, electrical and chemical signaling that allows for proper heart development, homeostasis and recovery from physiological stress or pathological injury (Bowers et al., 2010). The heart has variable ECM environment types including: 1) the basement membrane, that provides a cellular substrate and encompasses laminin, collagen IV, nidogen, perlecan and agrin, and 2) the interstitial matrix of the fibrous skeleton of the heart, that contains elastic fibers, fibronectin, collagens and proteoglycans (Derrick and Noël, 2021). The components of the ECM differ depending on the stage of heart development (Figure 2), and contribute to cell maturation and signaling processes, as well as provide mechanical support and stimuli to adjacent cells. Dysregulation of ECM components can lead to cardiac dysfunction, as well as progressive disease, demonstrating the important structural and regulatory roles of ECM. Transcriptomics and proteomics suggest an increase in collagen, laminin, and periostin content during cardiac development from the fetal to postnatal stage in mice (Notari et al., 2018; Silva et al., 2020; Derrick and Noël, 2021), followed by a decrease in fibronectin, hyaluronic acid, proteoglycans and agrin (Silva et al., 2020), suggesting a dynamic ECM regulation throughout development. In general, the prenatal and neonatal matrix is permissive for cell maturation and remodeling, while the relatively stiff ECM, apparent by P30, provides increased structural support and mechanical properties needed for increase cardiac demand. At the same time cardiomyocytes cease proliferation and undergo terminal maturation, which is suggestive of a relationship between cardiomyocyte cell cycle arrest and stiffening of the ECM in the postnatal period.

Cardiac function is dependent on the interactions of cardiomyocytes with ECM components produced by fibroblasts. During systole, the mature collagen fibrillar network transmits force to help maintain cardiomyocyte alignment while bearing wall stress (Fomovsky et al., 2010). In diastole, the collagen fibers uncoil as the ventricle fills and eventually completely straighten, protecting the cardiomyocytes from overstretching (Fomovsky et al., 2010). While elastin and multiple proteoglycans are also present, less is known about their contribution to the mechanics of the myocardium; these studies are long overdue to determine their roles in mechanics and function of the myocardium and their interaction with cellular components. Given our poor understanding of key processes in postnatal ECM changes, further studies are required in postnatal heart ECM to determine the key regulators and mechanisms involved in development, homeostasis and disease. Moreover, perturbation of expression of ECM components and associated changes in signaling pathways have been linked to congenital cardiac developmental anomalies as well as long term cardiac dysfunction.

### 4.1 Extracellular matrix composition and interactions in postnatal heart

The ECM represents a major regulator of intercellular processes in cardiac cells, in addition to providing integrity and mechanical support, responsible for functional and phenotypic changes (Bildyug, 2021). After birth, the heart undergoes extensive alterations in the ECM composition and structure to adapt to physiological requirements of the growing body. Major alterations after birth, include a decline in ECM molecules serving as morphogenic cues, such as fibronectin (FN), hyaluronic acid (HA), and proteoglycans, along with an increase in fibrillar collagens I and III and laminin, the structural molecules that help in maintaining the integrity of the cardiac ECM (Figure 2) (Silva et al., 2020). These alterations result in a more structured ECM, surrounding each cardiomyocyte in a honeycomb-like organization (Silva et al., 2020). The composition of cardiac ECM structural proteins is extremely important for pre- and postnatal heart development. For example, collagen XIV is highly expressed in the developing myocardium and plays an important role for growth and the structural integrity of the myocardium. At midgestation, mice deficient in collagen XIV had significant changes in mRNA level of collagens and remodeling enzymes within the ventricle myocardium; collagen mRNAs affected include significant increases in Col1a1, Col1a2, Col3a1, Col27a1 and Col12a1 and a reduction in Col6a1 and Col9a1 by E11.5, while only Col6a1 was affected in postnatal stages with an increase (Tao et al., 2012). Conversely, in postnatal stages, these mice experienced disorganized collagen-rich matrix, with fibrils abnormally dispersed throughout the interstitial space, and adult hearts resulted in defects in ventricular morphogenesis (Tao et al., 2012). Overall, developmental defects in Collagen XIV-deficient mice with compromised ECM, including increased in cardiomyocyte proliferation and a reduction in cardiac fibroblasts in association with increased apoptosis, leads to adult cardiac dysfunction at 3 months of age, including exacerbated thickening of the left ventricle wall (Tao et al., 2012). Furthermore, genetic ablation of other ECM components, including hyaluronan synthase, perlecan, versican, Col1, Col4, Col5, and FN, result in embryonic lethality with abnormal cardiac embryogenesis, while ablation of aggrecan, hapln1, glypican-3, Col2, fibulin1, or fibrillin1 is lethal perinatally (Hortells et al., 2019). Therefore, these ECM components and resulting structural networks, which undergo active remodeling after birth, are important for myocardial cell survival and cardiac function throughout life.

Integrins are fundamental components in the interaction between the ECM and the cardiomyocytes and fibroblasts. Their functions include the regulation of cellular phenotype in the developing and postnatal myocardium, adhesion, and migration (Ross and Borg, 2001). Integrins are non-covalently associated heterodimeric transmembrane receptors composed of *a* and *ß* subunits, which associate physically with fibronectin, the intracellular cytoskeleton, and other ECM proteins (Ross and Borg, 2001). Recently, there has been an interest in understanding to role of integrins, since transmembrane integrin receptors are critical for interactions between cells and the ECM. In addition, integrins are involved in mechanotransduction and converting the mechanical forces into biomechanical signals for a variety of cell types (Brancaccio et al., 1998). Major works on integrins role are mostly focused on development and disease using *in vitro* models. In cardiomyocytes, the main integrins are α_1_β_1_, α_5_β_1_, and α_7_β_1_, which are receptors for mainly collagen, fibronectin, and laminin, and these integrins are dynamically regulated during development (Bildyug, 2021). For example, cardiomyocyte integrins β_1D_ and β_1A_ are co-expressed in heart of E11 embryos, but the expression of β_1A_ declines as β_1D_ increases until it becomes the only β_1_ isoform present in cardiomyocytes a few days after birth (Brancaccio et al., 1998; Meagher et al., 2021). Expression of integrin α‐chains are also altered during heart development, where integrin subunit α_5_ is mainly expressed in fetal and neonatal cardiomyocytes, while in postnatal development, it is replaced by α_7_ integrin. Additionally, α_1_ and α_3_ subunits are also expressed in fetal and neonatal rat ventricular myocytes, but α_1_ chain is not expressed in adult hearts (Terracio et al., 1991; Bildyug, 2021). These individual *a* chains seem to be associated with splice variants of β_1_ integrin, specifically β_1D_, which is dominantly expressed in striated muscle and postnatal heart (Ross and Borg, 2001). The implications of these specific integrin changes for cardiac postnatal development myocardial disease states are not well-defined. Some insights have been provided by examination of intracellular mediators, such as pp125 focal adhesion kinase (FAK), small GTPases Rho or Rac, and cytoskeletal components such as talin (Ross and Borg, 2001). Additional support for the importance of integrins is provided by studies of Kindlin-2, an adaptor protein that binds to the integrin *ß* cytoplasmic tail to promote integrin activation, which is required to maintain integrin β_1D_ protein stability (Zhang et al., 2016). Postnatal deletion of Kindlin-2 from cardiac myocytes leads to progressive heart failure, demonstrating the importance of this protein in the normal heart function (Zhang et al., 2016). Definition of the specific roles of individual integrins in the postnatal heart is lacking, but available evidence supports important functions in mediating cardiomyocyte-ECM interactions. Notably, loss of adaptor proteins reinforces the importance of these mediators, and additional studies are needed to understand and potentially target these interactions.

### 4.2 ECM contributions to cardiomyocyte maturation and fibroblast activation in the postnatal heart

While the adult heart ECM is well studied, less is understood about neonatal and postnatal heart ECM composition and maturation. In the prenatal heart, fibroblasts produce the ECM when they first appear in development and continue predominantly after birth. In the embryonic heart (E9.5-E11.5), the thickness of the myocardium increases while cardiomyocytes proliferate and mature at the compact and trabecular layers, which is dependent on hyaluronan-rich cardiac jelly between endocardial and myocardial cell layers. Myocardial compaction and trabeculation are regulated by the transient expression of nephronectin and by the enzymatic degradation of the versican promoted by ADAMTSs (Silva et al., 2020). In the fetal heart (E14.5-E18.5), immature cardiac fibroblasts are dispersed in the primitive myocardium, surrounded by a loosely organized ECM composed of fibronectin, immature collagen, and proteoglycans. After birth, the ECM undergoes extensive remodeling characterized by a decrease in hyaluronic acid, FN, and proteoglycans, along with an increase in fibrillar collagen I and III, laminin, LOX, and periostin, (Figure 2), which influences cardiomyocyte proliferation. The fibrous skeleton of the mature heart evident 1–2 months after birth is composed primarily of an extensive fibrillar collagen network surrounding hypertrophic rod-shaped cardiomyocytes with highly organized sarcomeres and extensive electrical coupling (Silva et al., 2020).

Proteomic analysis of the postnatal heart demonstrates significant increases in collagen I, collagen III and laminin along with decreases in periostin and fibronectin in the weeks after birth (Figure 2) (Gershlak et al., 2013). *In vitro* experiments have demonstrated that fibroblasts and fibroblast-derived ECM components prevalent in the neonatal heart, such as fibronectin, collagen, periostin, heparin-binding epidermal growth factor, agrin and nephronectin, can induce cardiomyocyte proliferation and cytokinesis in early postnatal stages (Kuwabara et al., 2022a). Specifically, two embryonically enriched ECM proteins, SLIT2 and NPNT, have been implicated in postnatal heart maturation by promoting cytokinesis of postnatal cardiomyocytes *in vitro* and *in vivo* (Meng and Martin, 2020). Interestingly, neonatal fibroblast ablation leads to disrupted ECM composition, reduced collagen deposition, and decreased tissue stiffness in the heart, demonstrating an essential role for fibroblasts during perinatal heart development (Kuwabara et al., 2022a). Accordingly, changes in fibroblast ECM production in the perinatal heart coincide with heart maturation, highlighting the importance of understanding ECM composition and function.

Compelling evidence indicates that ECM stiffness is a decisive factor in postnatal heart development (Figure 2). Stiffness of the ECM regulates cell proliferation, dedifferentiation, migration, and fate (Garcia-Puig et al., 2019). ECM stiffness determines the cardiomyocyte cellular characteristics, where *in vitro* studies on neonatal rat cardiomyocytes cultured on polydimethylsioxane (PDMS) substrates (tunable stiffness ranging 2 MPa to 5 kPa) revealed that cardiomyocytes on rigid substrates (2 MPa) have aligned sarcomeres with larger, elongated, and triangular morphology (Yahalom-Ronen et al., 2015). Conversely, on softer substrates, the sarcomeres are disorganized with misaligned cytoskeletal structures, the cardiomyocytes are small, round and less polarized (Yahalom-Ronen et al., 2015). Furthermore, stiffer substrates promote cardiomyocyte differentiation and cell cycle arrest, while softer substrates allowed for cardiomyocyte dedifferentiation (Yahalom-Ronen et al., 2015), suggesting that the ECM content and organization play a major role in cardiomyocyte cell cycle activity.

### 4.3 ECM and cardiomyocyte regenerative capacity in the postnatal heart

ECM stiffness has been implicated in postnatal cardiac maturation and regeneration capacity. For example, decellularized zebrafish cardiac regenerative ECM is able to induce proliferation of both mice and human cardiac precursor cells, as well as significantly restore cardiac function and myocardial elasticity after MI (Chen et al., 2016). This suggests that both ECM protein composition and mechanical properties contribute to the ability of the heart to regenerate in zebrafish. In contrast, there is a transient neonatal regenerative period in mice. Apical resection (∼15% of the myocardium) in P1 mice is followed by complete regeneration of the myocardium 21 days post-resection (Porrello et al., 2011), with normal systolic function at 2 months compared to the sham controls. Similarly, an ischemic injury (myocardial infarction, MI) performed in P1 mice is immediately followed by significant tissue damage and altered cardiac function 24 h after injury (Haubner et al., 2012). Interestingly, 7 days post-MI, nearly complete regeneration was observed and 3-month follow-up of cardiac function showed complete restoration compared to the sham surgery controls. Notably, mice subjected to MI at P7.5 could not regenerate the myocardium P28.5 (21 days post-injury), but form a scar and exhibit decreased cardiac function (Haubner et al., 2012). Thus, the loss of regenerative capacity in neonatal mice coincides with cardiomyocyte cell cycle arrest and ECM maturation (Porrello et al., 2011; Haubner et al., 2012; Derrick and Noël, 2021). Since regenerative capacity declines soon after birth, gene expression profiling of P1 *versus* P2 mouse hearts was performed and demonstrated that the majority of the differentially expressed transcripts were related to ECM components, integrin binding, and structural components of the cytoskeleton (Notari et al., 2018). The hearts were also decellularized and tested for overall stiffness using AFM, which confirmed P2 hearts were ∼50% stiffer than P1 hearts (Notari et al., 2018). Therefore, there is a 7-day window after birth that mouse hearts can be regenerated, but as soon as the cardiomyocytes exit the cell cycle, that capacity is stopped, which coincides with significant ECM compositional changes, suggesting an interplay between ECM and cardiomyocyte maturation.

### 4.4 Mature and fibrotic extracellular matrix changes in development

The cardiac ECM undergoes significant changes in structure and protein composition in development, homeostasis, and post-injury remodeling (Figure 2). Interestingly, there are some shared characteristics of the ECM in the actively remodeling developing heart and adult cardiac injury response. During fetal and neonatal development, when cardiomyocytes are proliferating and capable of regeneration, the main ECM proteins are fibronectin, fibrillin-1, fibrillin-2, collagens IV and VI, laminin, agrin, hyaluronic acid, proteoglycans, and perlecan (Notari et al., 2018; Silva et al., 2020; Derrick and Noël, 2021). Soon after birth, the composition of the ECM changes switching to a more stiff and less regenerative environment, with increased fibrillar collagens, laminin, periostin and decreased fibronectin, hyaluronic acid, proteoglycans and agrin (Silva et al., 2020). Once the heart is matured, adult heart repair after injury includes increased expression of fetal-associated ECM, especially fibronectin and hyaluronan, but the adult cardiomyocytes are unable to proliferate, resulting in formation of a collagen-rich scar and interstitial fibrosis (Silva et al., 2020). Although the process of cardiac repair in adults starts with similar ECM molecules as the developing heart, such as induction of fibronectin and tenascin C also seen in regeneration, once cardiomyocytes have exited the cell cycle, upregulation of these ECM proteins no longer induces proliferation, but does lead to an accumulation of a collagen-rich ECM in the myocardium and formation of a stiff scar (Silva et al., 2020). ECM proteins including fibrin, fibrinogen and fibronectin, as well as MMPs, are increased, and fibroblasts start to secrete other ECM molecules such as proteoglycans, hyaluronan, and versican (Silva et al., 2020). Soon after, the collagen content increases and lysyl-oxidase (LOX) is upregulated, inducing collagen cross-linking (Silva et al., 2020), leading to the formation of a scar without functional contractile properties. This ultimately leads to a stiffer heart, electrical conduction impairment, and heart failure (Miragoli et al., 2007; Swan et al., 2018; Silva et al., 2020). Overall, the neonatal and injured ECM share common features, but ultimately the normal healthy fibrous ECM and injured scar have different ECM proteins with significant implications for heart function and disease.

## 5 Discussion

Cellular crosstalk between cardiac fibroblasts and cardiomyocytes embedded in a dynamically remodeling ECM is essential to the postnatal development of the heart. Notably, during this time period, cardiomyocytes undergo cell cycle arrest and transition to hypertrophic growth with increased contractility, oxidative capacity, and energy production (Borg et al., 1981), while fibroblasts become are activated, proliferative and synthesize specialized ECM. At the same time, the ECM components transition from a loosely organized soft proteoglycan/hyaluronan-rich matrix to a stiffer fibrillar collagen matrix necessary for mature cardiac function and force generation of the postnatal ventricles. Additionally, transcriptomic and proteomic studies demonstrate heterogeneity of fibroblast populations, mechanisms of cardiomyocyte maturation, and changes in multiple cell signaling pathways, in the context of ECM protein changes. Together, the interactions of multiple cardiac cell lineages within the dynamic microenvironment of the postnatal heart offers clues to understanding the specific crosstalk mechanisms that underlie fibroblast and cardiomyocyte specific changes in development with implications for disease.

Current sequencing technologies have allowed us to deepen our understanding of cardiac cell diversity, particularly fibroblast cell heterogeneity. Notably, developmentally activated fibroblasts in the context of the neonatal heart share some characteristics, such as periostin expression, with adult cardiac fibroblasts after injury, but do not completely transition to a myofibroblast state as defined by αSMA/ACTA2. Thus, it may be possible to induce a healthy cardiac ECM *versus* pathologic fibrotic or scar ECM through manipulation of fibroblast differentiation/activation states. Moreover, genetic manipulation of cardiac fibroblasts through reprogramming (Qian et al., 2012) or lineage targeted delivery of antifibrotic chimeric antigen receptor (CAR) T cells show potential for new heart failure therapies (Rurik et al., 2022). Thus, the increased understanding of fibroblast subtypes and interactions with cardiomyocytes is critical for improved cell-specificity of targeted *in-vivo* manipulations, as well as furthering our understanding of the specific changes fibroblasts undergo as a response to environmental stimuli in development and disease. Particularly interesting is the discovery of the different roles for periostin + fibroblasts in postnatal heart development compared to cardiac injury response (Hortells et al., 2020). In addition, single cell and cell type-specific sequencing technologies allow for the identification of potential crosstalk mechanisms between various types of cells. However, a major limitation to the current field is the difficulty in validating specific bioinformatically identified crosstalk mechanisms *in vivo*. Ongoing studies are likely to be informative in this area.

An additional attractive therapeutic approach is targeting the composition and mechanical properties of the cardiac ECM. The healthy ECM of the postnatal period, characterized by a hyaluronic acid, fibronectin, and proteoglycans, supports regenerative cardiac growth, while the fibrous skeleton of the mature heart, composed primarily of fibrillar collagen, is required for effective cardiac output and physiology throughout life. However, too much fibrillar collagen leads to fibrotic remodeling, decreased cardiac contractility and scarring in disease. Thus control of cardiac fibroblast proliferation, maturation status and ECM production is important, and the correct balance of collagens, laminin, LOX, and periostin ECM components must be controlled in any manipulation of fibroblast activation or cardiomyocyte maturation status (Silva et al., 2020). Importantly, the composition of the healthy ECM varies greatly from that of an unhealthy ECM. For example, following cell death, the ECM is infiltrated by inflammatory cells and secretion of proteins that modulate cell function, promote matrix assembly, and protect the myocardium as adverse remodeling begins and formation of a collagen-rich scar leading to fibrosis takes place (Silva et al., 2020). Increased understanding of ECM components and remodeling mechanisms that contribute to a production of a healthy cardiac matrix *versus* pathologic scarring and cardiac dysfunction may lead to important new therapies for heart failure and fibrotic disease.

In summary, cardiomyocyte-fibroblast-ECM interactions are the foundation for heart development, homeostasis, and disease. Shifts in any of these components can lead to detrimental changes, as they are all constantly communicating *via* secreted ligands and mechanical signaling. For many years, the concept that the complex network of intracellular circuits of communication is essential for proper maturation and maintenance of a healthy heart has been widely unappreciated but not fully understood. Due to recent technologies, more clues have been obtained into the importance of coordinated functions of different cell populations in heart development and how the specific mechanisms underlying these interactions could be leveraged as therapeutics for heart disease. Further, new insights into cellular and ECM crosstalk mechanisms in the heart are still being discovered, and this is an active area of research by many investigators in the field.
